# Using cell fate attractors to uncover transcriptional regulation of HL60 neutrophil differentiation

**DOI:** 10.1186/1752-0509-3-20

**Published:** 2009-02-18

**Authors:** Albert C Huang, Limei Hu, Stuart A Kauffman, Wei Zhang, Ilya Shmulevich

**Affiliations:** 1 Molecular and Cellular Biology Program, University of Washington, Seattle, Washington, DC, USA; 2 Institute for Systems Biology, Seattle, Washington, DC, USA; 3 Cancer Genomics Laboratory, University of Texas M.D. Anderson Cancer Center, Houston, Texas, USA; 4 Institute for Biocomplexity and Informatics, University of Calgary, Calgary, Alberta, Canada

## Abstract

**Background:**

The process of cellular differentiation is governed by complex dynamical biomolecular networks consisting of a multitude of genes and their products acting in concert to determine a particular cell fate. Thus, a systems level view is necessary for understanding how a cell coordinates this process and for developing effective therapeutic strategies to treat diseases, such as cancer, in which differentiation plays a significant role. Theoretical considerations and recent experimental evidence support the view that cell fates are high dimensional attractor states of the underlying molecular networks. The temporal behavior of the network states progressing toward different cell fate attractors has the potential to elucidate the underlying molecular mechanisms governing differentiation.

**Results:**

Using the HL60 multipotent promyelocytic leukemia cell line, we performed experiments that ultimately led to two different cell fate attractors by two treatments of varying dosage and duration of the differentiation agent all-trans-retinoic acid (ATRA). The dosage and duration combinations of the two treatments were chosen by means of flow cytometric measurements of CD11b, a well-known early differentiation marker, such that they generated two intermediate populations that were poised at the apparently same stage of differentiation. However, the population of one treatment proceeded toward the terminally differentiated neutrophil attractor while that of the other treatment reverted back toward the undifferentiated promyelocytic attractor. We monitored the gene expression changes in the two populations after their respective treatments over a period of five days and identified a set of genes that diverged in their expression, a subset of which promotes neutrophil differentiation while the other represses cell cycle progression. By employing promoter based transcription factor binding site analysis, we found enrichment in the set of divergent genes, of transcription factors functionally linked to tumor progression, cell cycle, and development.

**Conclusion:**

Since many of the transcription factors identified by this approach are also known to be implicated in hematopoietic differentiation and leukemia, this study points to the utility of incorporating a dynamical systems level view into a computational analysis framework for elucidating transcriptional mechanisms regulating differentiation.

## Background

The process of cellular differentiation is central to our understanding of the nature of multicellular living systems, their stability in a changing environment, and how such systems fail in diseases, such as cancer [1,2]. This developmental process of individual cells in a multicellular organism committing to their specialized phenotypic characteristics is temporally coordinated by a complex dynamical system comprised of large numbers of interacting genes and their products [3-5]. Not surprisingly, dynamical systems theory has been used to study cell differentiation [6-8].

Despite its tremendous importance, there is very little accumulated knowledge of the process of differentiation from a systems perspective and of the role of molecular programs involved in this process. Even for an agent that causes differentiation to a common recognizable state, we do not know whether the cells, as manifestations of the underlying dynamic bio-molecular systems, always follow common or different molecular paths (or system state trajectories). In the latter case, we also do not know which of those paths is the most stable and least reversible.

Since a cell's phenotype and behavior are largely determined by the activities of the genes and proteins constituting a genetic network, it follows that the rules of interactions between these elements translate directly into rules of cellular behavior. That is, the enormous state space of a genetic network (i.e., the space of all possible configurations activities of the constituent elements) becomes reduced into a relatively small number of trajectories and steady states (attractors) of the dynamical system. Kauffman postulated that these attractor states in model networks correspond to the cell types in multicellular organisms [9,10], and the process of differentiation corresponds to a trajectory (in the state space) leading into one of the attractors. The cellular fate is thus determined by the attractor in which the genetic network eventually ends up; this can, to a large extent, be controlled by appropriate external stimuli that place the system into different initial states. It is important to note that many trajectories ensuing from such different initial states can flow to a common attractor and thus constitute its basin of attraction.

Consider that small molecule chemicals, such as dimethyl sulfoxide (DMSO) and a host of others can induce cell differentiation in a variety of cell systems along with concomitant cellular properties [11-15]. This rather amazing fact implies the pre-existence of cellular fates that need only be selected by means of external stimuli rather than created by specific molecular events. This 'selection' of cell fates occurs by means of the inherent nature of the dynamical system to flow to an attractor when placed in some initial transient state and thus, differentiation is a process of selecting a particular attractor in a genetic regulatory network. This view has been supported experimentally by Huang *et al*. using genome-wide mRNA expression profiling [16] as well as by means of analyzing cell fates in response to generalized physical stimuli, such as cell distortion [17]. For a more extended discussion on this topic, see [10].

The homeostatic stability of a differentiated cell is a consequence of the underlying stability of the attractor – 'nearby' states, which may occur as a consequence of natural environmental variation, simply flow back to the attractor. It is known that normal cells have a balanced state of proliferation and differentiation, resulting in homeostatic stability [18,19]. A block of normal differentiation and abnormal reversal of differentiation (sometimes called de-differentiation) [20] are believed to be some of the hallmarks of cancer [21]. Accordingly, therapeutic strategies have been designed to facilitate cancer cells to reenter the differentiation program, often termed differentiation therapy [22,23].

The success of such therapeutic strategies depends on our ability to systematically determine appropriate molecular 'lever points', the perturbations of which place the biomolecular system into states that are poised to differentiate. Indeed, such a strategy corresponds to placing the system in a state by means of a stimulus, such as a therapeutic agent, and allowing the system to naturally flow toward an attractor that corresponds to the desired cellular endpoint [24-26]. To identify such targets for intervention, it is necessary to characterize the underlying molecular mechanisms, such as transcriptional regulatory networks, governing the process of differentiation. Systems biology approaches, which are predicated on global measurements and data integration, are now beginning to reveal transcriptional machinery underlying complex biological processes [27-30]. The rationale behind our study was to explore whether the aforementioned systems-level view of cell fates as attractors and differentiation as a route toward an attractor, when coupled with computational systems biology approaches, is informative for elucidating the transcriptional regulatory mechanisms governing differentiation.

To this end, we have selected a well-established differentiation model, human promyelocytic leukemia cells (HL60) originally isolated by Dr. Steven Collins from a 37-year-old female acute promyelocytic leukemia (APL) patient [31]. The HL60 is a multi-potent cell line that can be stimulated to differentiate using a variety of chemical agents, including DMSO [32], all-*trans*-retinoic acid (ATRA) [33], 1,25*α*-dihydroxyvitamin *D*_3 _[34], 12-O-tetradecanoylphorbol 13-acetate (TPA) [35], and granulocyte macrophage colony-stimulating factor (GM-CSF) [36]. With the addition of ATRA, the HL60 cells differentiate into neutrophils, while displaying the early differentiation marker, CD11b, which begins to be expressed within one day of treatment [37]. Although there are others, CD11b is an early differentiation marker, which allows one to capture the initial stage of the process. The CD11b+ differentiated HL60 cells can be stained with fluorescent-labeled anti-CD11b antibody and easily recognized by commonly used flow-cytometry methods and isolated by flow-sorting for further culturing and experimentation, as we have done here. The HL60 system was also used by Huang *et al*. [16] to demonstrate the correspondence between cell fates and high-dimensional attractor states of the underlying genetic network.

One could reason as follows. If we could place the HL60 into a state from which the system would dynamically flow towards the "neutrophil" attractor, as demonstrated by Huang *et al*., then the genes that show altered behavior along the time-course trajectory relative to unstimulated controls could be hypothesized to be implicated in the neutrophil differentiation process. This, of course, may be the case, though the interpretation is confounded by the possibility that the genes exhibiting altered behavior are responsive to the particular mechanisms activated by the stimulus used, such as ATRA. Indeed, Huang *et al*. also confronted this conceptual difficulty when they compared trajectories from ATRA-treated and DMSO-treated HL60 cells, finding that certain genes may behave differently simply as a result of different stimuli activating different biological pathways, while many other genes dynamically converge towards a common attractor, despite the system flowing from distinct starting states corresponding to ATRA and DMSO treatments [16]. To identify genes that are not stimulus dependent, but are involved in the process of neutrophil differentiation, one could then use only one treatment, but in a way that allows one to alter cellular fate, namely, terminal differentiation into neutrophils or reversion back to the undifferentiated state.

The HL60 was shown to exhibit such behavior in two separate studies both demonstrating that this differentiation process contains at least two steps in which a precommitment stage precedes the decision to differentiate. Yen *et al*. observed that with continuous exposure of ATRA at a high concentration, the HL60 proceeds through differentiation, but upon removal of the stimulus, the HL60 falls back to the undifferentiated state [38]. By analogy, such a precommitment stage corresponds to a gradually sloping plateau between a valley and a mountain such that a ball sitting on this plateau would roll down into the "undifferentiated" valley in the absence of additional energy necessary to make it over the "terminally differentiated" mountain. More recently, Chang *et al*. reported a population of "primed," undifferentiated CD11b- cells upon exposure to a low dose DMSO [39]. Though these cells are negative with respect to the CD11b marker, thus considered to be "undifferentiated," upon encountering a second dose of DMSO stimulation, they exhibited an increased rate of differentiation, suggesting that the first low dose DMSO had placed them in a "primed" intermediate differentiated state.

We thus decided to determine two different treatments, both with ATRA, but with different concentrations and incubation times such that the two cell populations corresponding to these treatments would be poised at the same stage of differentiation (precommitment), but so that one population follows through to the terminally differentiated neutrophil attractor, while the other reverts back by dynamically flowing towards the undifferentiated state. The genes that would exhibit different behavior between these two trajectories would then be potentially implicated in the differentiation process.

To identify two such precommitment states, we used the percentage of CD11b+ cells at the end of a particular treatment as a measure of the stage of differentiation. We performed 80 ATRA treatments consisting of 8 doses (0.0005 *μ*M to 1 *μ*M) and 10 durations (4 to 13 days) in triplicate and measured percentages of CD11b+ cells, relative to an isotype antibody control, using FACS analysis. Consider loci in the two-dimensional dose × duration stimulus space, where all points on a particular locus correspond to a constant fraction of CD11b+ cells. Thus, two cell populations on the same locus can be said to be at the same "stage" of differentiation at least as it pertains to CD11b. We chose two such populations, one with a higher dose and a shorter duration and the other with a lower dose and a longer duration, such that the cells treated with the higher dose proceeded with differentiation into neutrophils while the cells treated with the lower dose reverted back to the undifferentiated state, despite both populations exhibiting the same percentage of CD11b+ cells at the end of their respective treatments. The cells were live-sorted, cultured in fresh media, and profiled every 24 hours with microarrays for five days in triplicate. This additional Fluorescence Activated Cell Sorting (FACS) step mitigates the confounding effects of cellular heterogeneity due to subpopulations that do not initiate the differentiation program (i.e. CD11b- cells). In this manner, the gene expression programs of the two cell populations, one differentiating and one reverting, could be analyzed using computational approaches.

We defined a criterion to identify genes whose behavior over time exhibits a divergence between the two treatments. It is these genes that are hypothesized to be involved in the differentiation process. We analyzed the promoters of these genes and found that they are overrepresented with known transcription factors functionally linked to myeloid differentiation, cell cycle, and development. This study points to the utility of incorporating a systems-level view of global dynamics, as distinguished from the dynamics or kinetic behavior of the individual elements of a system, into a computational analysis framework that can be used for studying transcriptional regulatory mechanisms governing a complex biological process such as differentiation.

## Results and discussion

### Two comparable dosage/duration treatment combinations lead to different macroscopic cell fate attractors

Our first goal was to determine two dosage/duration stimulation conditions that yield comparable stages of differentiated HL60 cells, with one condition ultimately leading to neutrophil differentiation and the other reverting back to the undifferentiated state. In other words, we sought to identify two perturbations that place the ATRA-treated cells in two different basins of attraction or initial states leading to two different attractors – the promyelocyte attractor and the neutrophil attractor. This information allows us to culture large quantities of the HL60 cells under these conditions and isolate mRNA for time course experiments to examine the set of genes differentially expressed between these treatments that could explain the differences in their eventual cell fates.

**Figure 1:**
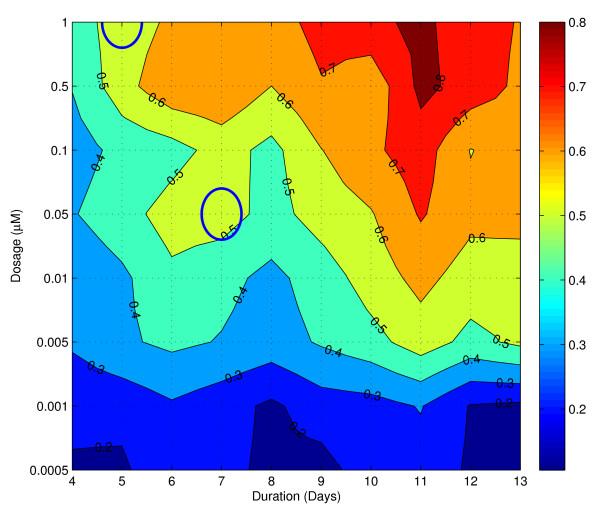
**Contour plot of the percentage of CD11b+ cells after ATRA treatment**. The x-axis represents the duration of ATRA treatment (Days). The y-axis represents the dosage of ATRA treatment (*μ*M). The ovals represent the 1 *μ*M/5 Days and 0.05 *μ*M/7 Days dosage/duration culture conditions utilized for gene expression analysis. An increase in dosage or duration leads to an increase in CD11b+ cells.

To achieve this goal, we set up a two-factor dosage and duration experiment with eight and ten levels respectively, ranging from 0.0005 *μ*M to 1 *μ*M with 4 to 13 days of treatment. We used a well-established early marker for neutrophil differentiation, CD11b, as our surrogate for 'differentiated' and 'undifferentiated' state [37]. We measured the CD11b expression for each experimental condition and calculated the percentage of HL60 cells that are CD11b+ by comparing to the untreated samples. Under this construction, the percentage of CD11b+ cells becomes a proxy for the developmental stage of neutrophil differentiation on a population level. The result of this dosage and duration experiment is summarized and displayed in a contour plot (Figure 1), showing a general trend – as the dosage or duration of ATRA treatment increases, the percentage of CD11b+ cells also increases. The treatment combinations, 0.5 *μ*M/11 Days produced the highest percentage of CD11b+ cells at 82.7%. As expected, this result conforms with our general intuition regarding ATRA treatment, that an increase in the dosage or the duration of treatments results in an increase in the percentage of differentiated CD11b+ cells. See Additional File 1 for the percentages of CD11b expression of the various treatments.

From the contour plot, we identified two treatments that both gave rise to 54 percent of CD11b positive cells, namely, 1 *μ*M/5 Days and 0.05 *μ*M/7 Days. These two treatment combinations were picked because they produced similar levels of positive CD11b expression, yet one treatment is of higher dosage with shorter duration, while the other is of lower dosage with longer duration. We grew the HL60 cells under those conditions and isolated the CD11b+ population of these cells by FACS and re-cultured these cells in fresh, ATRA-free RPMI-1640 media. We collected the re-cultured cells and isolated the mRNA for whole-genome expression analysis each day for five days. At the end of this period, we also collected cells from both treatments for Wright-Giemsa staining, a histological method that could be used to determine hematopoietic cell types based on cell morphology. The 0.05 *μ*M/7 Days treatment resembles that of the untreated HL60 cells, with clear visible nucleoli and large nuclear to cytoplasm ratios, suggesting a reversal of cell fate back to the undifferentiated HL60 state; whereas the 1 *μ*M/5 Days treatment shows morphology resembling that of differentiated neutrophils, with characteristic decreased nuclear to cytoplasmic ratio, and convolution and segmentation of the nuclei, suggesting a completion of cell fate toward the differentiated neutrophil attractor. Our observation is in accordance with the notion of a "precommitment" state previously described [39-41], whereupon the removal of the stimulating agent, the HL60 can revert back to the undifferentiated state. Taken together, we have established a system where we identified two perturbations that place the HL60 cells in different basins of attraction, leading to different eventual macroscopic cell fates.

### A subset of the genes leading to different cell fate attractors exhibit a divergent expression pattern

To understand how the macroscopic cell fates that we observed could have arisen from these two perturbation conditions, we analyzed the gene expression profiles of the treated HL60 cells. We reasoned that we had placed the treated HL60 cells in their perspective basins of attraction when we re-cultured the sorted CD11b+ cells from these two treatments in ATRA-free media. Hence, the gene expression trajectories reflected the natural consequences of placing the HL60 cells in these specific parts of the genomic expression space. Therefore, when we looked at the gene expression profiles of differentially expressed genes between the two trajectories, the pattern we observed could potentially explain the observed macroscopic cell fate.

**Figure 2:**
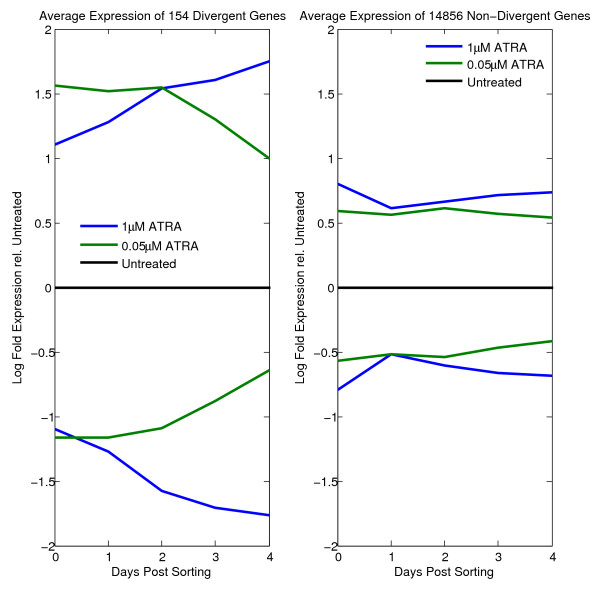
**Average mRNA gene expression of the differentially expressed genes**. The left panel shows the divergent genes. The right panel shows the non-divergent genes. The upper parts of the figures represent the genes that are up-regulated in both 1 *μ*M/5 Days and 0.05 *μ*M/7 Days treatments relative to untreated controls. The lower parts represent the genes that are down-regulated in both 1 *μ*M/5 Days and 0.05 *μ*M/7 Days treatments relative to untreated controls.

Interestingly, while the majority of the differentially expressed genes (relative to untreated controls) exhibit a flat and unchanging average gene expression profie under both (1 *μ*M/5 Days) and (0.05 *μ*M/7 Days) treatment conditions, there is a small subset of the genes (154, using our criterion) that exhibit a divergent gene expression profile. That is, after the high dosage/short duration treatment (1 *μ*M/5 Days), their expression levels deviate further and further away from their levels under the untreated ATRA condition, whereas their expression levels after the low dosage/long duration treatment (0.05 *μ*M/7 Days) converge toward the gene expression levels of those under untreated ATRA condition (Figure 2). These divergent genes can be separated further into two distinct classes, the up-regulated, and the down-regulated genes. The up-regulated (respectively, down-regulated) genes are the ones that have elevated (respectively, repressed) expression under both high dosage/short duration and low dosage/long duration treatments relative to their expression under the untreated ATRA condition. In both cases, this display of differential expression behavior reflects the macroscopic cell fate observed, namely that the HL60 cells from the high dosage/short duration treatment continue toward differentiation whereas cells subjected to low dosage/long duration treatment revert back toward the undifferentiated state. We hypothesized that these divergent genes participate in the selection of a particular attractor from a set of pre-existing ones. See Additional File 2 for a list of the divergent genes as well as magnitude of divergence.

### Divergent genes promote cellular differentiation and repress cell cycle progression

After we identified the set of divergent genes and their unique gene expression patterns, we set out to investigate their known biological functions, with the goal of elucidating how these genes coordinate the transition of the HL60 cells from the promyelocyte attractor into the neutrophil attractor. In particular, cellular differentiation processes frequently entail an up-regulation of genes involved in specialization while simultaneously down-regulating genes related to proliferation and cell cycle [18,19].

Indeed, a number of the up-regulated divergent genes are involved in the activation and specialization of neutrophils. At the top of the up-regulated divergence gene list is ankyrin repeat and SOCS box-containing 2 (ASB2), which is known to be a retinoic acid-response gene and a binding target of the promyelocytic leukemia retinoic acid receptor-alpha (RAR*α*) oncogenic protein [42]. When ASB2 is expressed in leukemia cells, it inhibits growth and furthers myelocytic commitment, precisely as seen in the HL60 cell differentiation model system. Inherent to the process of neutrophil activation are genes promoting the homing and migration of neutrophils to the sites of inflammation. Examples of these genes among this list are orosomucoid 1 (ORM1), interleukin 8 receptor beta (IL8R*β*), and vanin2 (VNN2). ORM1 is highly expressed during acute inflammation and has been suggested as a signaling molecule that binds to L-selectin on the neutrophil cell surface to allow neutrophils to enter secondary lymphoid tissues via the high endothelial venules [43]. IL8R*β*, also known as CXCR2, is a receptor of interleukin 8 (IL8) and facilitates neutrophil migration to the site of inflammation [44]. Recently, its ligand was identified to be the cytokine, macrophage migration inhibitory factor (MIF) [45]. Finally, expressed mainly on human neutrophils [46] and anchored on the cell surface by glycosylphosphatidylinositol (GPI), VNN2 physically associated with *β*_2 _integrin (CD11b), the neutrophil differentiation marker for our study [47].

Besides neutrophil activation, response to inflammation is another process in which several of the up-regulated divergent genes including leukocyte immunoglobulin-like receptor subfamily B member 3 (LILRB3) and NOD9 (NLR X1) participate. Expressed LILRB3 protein binds to the MHC class I molecules on antigen-presenting cells to control inflammatory responses and cytotoxicity by transducing a negative signal that inhibits immune response and limits autoreactivity [45]. NOD9 is a member of the NLR family known to recognize microbial molecules that activate inflammatory caspases, causing cleavage and activation of inflammatory cytokines [48]. Collectively, these up-regulated divergent genes promote requisite activities of activated neutrophils.

The list of down-regulated divergent genes, on the other hand, contains many genes necessary for the progression of cell cycle. We found members of the well-known cyclin and cell division cycle (CDC) gene families [49] including cyclin d2 (CCND2), cyclin E1 (CCNE1), cell division cycle 2 (CDC2), cell division cycle 7 (CDC7), CDC28 protein kinase regulatory subunit 1B (CKS1B), and cell division cycle associated 5 (CDCA5). CCND2 forms a complex with cyclin dependent kinase 4 (CDK4) [50] and regulates cell cycle G0/G1 to S transition [51]. Similarly, CCNE1 is necessary for the progression of G1 to S transition. It interacts with cyclin dependent kinase 2 (Cdk2) to phosphorylate the target genes nuclear protein ataxia-telangiectasia locus (NPAT) and nucleophosmin, critical components of cell proliferation and DNA replication, respectively [52]. CDC2 encodes a Ser/Thr kinase and is the catalytic subunit of M-phase promoting factor (MPF), critical for G1/S and G2/M transitions [53]. CDC7 encodes a kinase that is essential for DNA replication as well as the transition between G1/S phase [54]. CKS1B binds to cyclin A for targeted degradation and passage through the spindle checkpoint [55]. CDCA5, also known as sororin, controls the separation of sister chromatids during mitosis by stabilizing centromeric cohesin [56]. In addition to the cyclin and CDC gene families, other genes integral to cell cycle progression were also found. TTK protein kinase (TTK) is a cell cycle-regulated kinase with maximal activity during M phase, localizing to kinetochores [57]. It is required for centrosome duplication and normal progression of mitosis [58]. Kinesin family member 20A (KIF20A) accumulates in mitotic cells where it localizes to the midzone of the spindle during anaphase, and to the cleavage furrow and midbody during telophase, essential for cytokinesis to proceed [59]. Interestingly, KIF20A is a target of polo like kinase 1 (PLK1), a protein that we also identified as a divergent gene. Together, they are necessary for proper spindle assembly and function during anaphase and telophase of the cell cycle [60]. Jointly, the down-regulated divergent genes suppress the HL60 cells from progressing through the cell cycle.

To complement our literature-search approach to understanding the functions of these up and down-regulated divergent genes, we examined them separately in the context of Gene Ontology (GO) [61] enrichment analysis using GoMiner [62]. The 154 divergent genes include 48 up-regulated and 106 down-regulated divergent genes out of a total of 30729 unique genes on the Agilent array. However, at the relative conservative *p*-value and FDR level of 0.05 and due to a relatively small number of up-regulated divergent genes, we are only able to detect statistical significance of enrichment for the down-regulated divergent genes. The GO enriched results for the down-regulated divergent genes are listed in Table 1.

**Table 1:** Enriched GO categories of down-regulated divergent genes.

GO Terms and Description	Total Genes	Changed Genes	-log_10_(*p*)	FDR
(Biological Process)				
GO:0007049 Cell cycle	597	14	5.7044	0
GO:0051301 Cell division	187	7	4.2512	0.005
GO:0022402 Cell cycle process	334	9	4.22	0.003
GO:0045739 Positive regulation of DNA repair	5	2	3.5589	0.03
GO:0051726 Regulation of cell cycle	248	7	3.4893	0.026
GO:0000082 G1 S transition of mitotic cell cycle	30	3	3.2793	0.032
GO:0009132 Nucleoside diphosphate metabolic process	7	2	3.2397	0.036
GO:0051329 Interphase of mitotic cell cycle	76	4	3.1561	0.035
GO:0006259 DNA metabolic process	373	8	3.1266	0.031
GO:0006282 Regulation of DNA repair	8	2	3.1162	0.039
GO:0051325 Interphase	79	4	3.0926	0.036
GO:0006396 RNA processing	298	7	3.0138	0.038
GO:0009262 Deoxyribonucleotide metabolic process	9	2	3.0086	0.042
GO:0007059 Chromosome segregation	38	3	2.9748	0.04

This table contains the enriched GO categories of the down-regulated divergent genes. The first column includes the GO terms and description. The second column contains the total number of genes for that given category on the Agilent microarray. The third column shows the number of down-regulated divergent genes for that category. The fourth column lists the -log_10 _probability associated with that categories. The fifth column contains the false discovery rate.

### Transcription factor binding site enrichment in promoters of divergent genes

We suspected that there may be common regulatory mechanisms that control the expression of both up and down-regulated divergent genes to select for the neutrophil cell fate attractor and to efficiently activate the necessary biological processes. One common mechanism of controlling gene expression is through the regulatory actions of transcription factors; therefore, we set out to search for enriched transcription factor binding sites (TFBS) in the promoter regions of both the up and down-regulated divergent genes simultaneously. We used a background model composed of upstream 2 kb promoter sequences of all known genes and compared them to the upstream promoter sequences of the divergent genes by calculating the log likelihood ratio score to find the putative binding sites. Table 2 contains the sorted top 15 enriched transcription factor binding sites.

**Table 2:** Top 15 enriched transcription factor binding sites

TF Name	Biological Process	Functional Category	Reference
1) C/EBP*α*	Acute myeloid leukemia	Tumor Progression	[63]
2) HLF	Acute lymphoblastic leukemia	Tumor Progression	[67]
3) PAX6	Eye development	Development	[90]
4) TAL1/SCL	Lymphocytic leukemia	Tumor Progression	[91]
5) HOXA4	Chronic lymphocytic leukemia	Tumor Progression	[73]
6) CDP/CUTL1	Mediate cell cycle progression	Cell Cycle	[92]
7) AML/RUNX1	Acute myeloid leukemia	Tumor Progression	[93]
8) TBP	Required for RNA Pol II	General Transcription	[94]
9) MEIS1	Acute myeloid leukemia	Tumor Progression	[79]
10) OCT-1/POU2F1	Regulates cyclin D1 w/STAT5	Cell Cycle	[95]
11) RAR-RXR	Acute promyelocytic leukemia	Tumor Progression	[81]
12) POU3F2	Neuronal development	Development	[96]
13) FOXO4/AFX	Mixed lineage leukemia	Tumor Progression	[82]
14) SOX9	Chondrogenesis	Development	[97]
15) POU1F1	Pituitary hormone secretion	Development	[98]

This table contains the top 15 enriched transcription factors binding sites found in the promoters of divergent genes, with literature references that link these factors to general functional categories of tumor progression, cell cycle, and development. The first column includes the transcription factor name. The second column contains the biological processes with which these factors are associated. The third column lists the general functional categories of these factors. The fourth column points to the literature references for these factors.

Functionally, these enriched TFBSs can be broken down into rough categories of tumor progression, cell cycle, development, or general transcription, which are processes actively engaged by the HL60 neutrophil differentiation model system. Let us consider the striking roles played by the transcription factors in tumor progression. We find TFBSs for C/EBP*α*, HLF, TAL1, HOXA4, MEIS1A, RAR-RXR, and FOXO4, all of which, when disregulated, have been shown to lead to leukemia. C/EBP*α *is crucial for the process of granulopoiesis, and its aberrant expression in acute myeloid leukemia is well-studied [63-66]. Conditional expression of C/EBP*α *leads to neutrophil differentiation while dominant-negative expressions of C/EBP*α *are found in AML patients [63]. HLF, together with E2A, forms a chimeric E2A-HLF transcription factor protein due to a translocation mutation t(17;19)(q21;p13) which occurs in a subset of acute lymphoblastic leukemias [67-69]. TAL1 is essential for early stage embryonic hematopoiesis; erythroid differentiation is associated with increased TAL1 expression, while myeloid differentiation is associated with decreassed TAL1 expression [70]. A TAL1 translocation mutation t(1;14)(p32;q11) is observed in 3% of patients with T cell acute lymphoblastic leukemia [71]. HOX genes are an important transcription factor family for hematopoiesis, the different family members of which are required to specify particular stages of hematopoietic development [72]. Further, HoxA4 promoter hypermethylation has recently been linked to mutational status in chronic lymphocytic leukemia [73]. AML/Runx1 is also linked to hematopoiesis and leukemic tumor progression [74]. It is the DNA binding element of the core binding factor (CBF) transcription complex and is required for hematopoiesis as shown in knock-out mouse studies [75]. Mutations of RUNX1 have been identified in familial platelet disorder (FPD) along with a congenital predisposition to the development of acute myeloid leukemia (AML) [76]. MEIS1, another protein with enriched TFBS, cooperates with both HOXB3 and HOXA9 to induce the transcription factor AML [77,78] by down-regulating its expression through promoter hypermethylation in a subset of AMLs [79]. The RAR-RXR heterodimer TFBS is also enriched in the promoter regions of our divergent genes. Since the HL60 neutrophil differentiation is induced through the actions of retinoid acid, it is reasonable that we observed an enrichment of retinoid acid receptor binding sites. Though it is well-established that the chimeric fusion protein from RAR-PML translocation mutation, t(15;17), is frequently associated with acute promyelocytic leukemia [80], methylation analysis of the RAR*α *promoter further cements its involvement [81]. Finally, FOXO4 has also been linked with acute leukemias. A translocation mutation t(X;11)(q13;q23), which fuses it with the gene MLL, was observed and cloned [82]. In summary, the list of enriched TFBSs recapitulates many important regulators of hematopoiesis, which are intimately tied to leukemia pathology, illustrating the potential utility of such systems-level experimental designs.

## Conclusion

In this study, we perturbed the HL60 cells into the basins of attraction of two distinct cell fate attractors using two different ATRA dosage/duration treatments such that both cell populations are poised at the same stage of differentiation. By tracking the gene expression changes en route to these cell fates, we found a subset of the differentially expressed genes that exhibited a divergent gene expression pattern, hypothesized to correspond to the observed macroscopic cell type phenotype. Literature searches identified the possible functions of these divergent genes to be involved in promoting neutrophil differentiation and repressing cell cycle progression. Analyses of the promoter sequences of the divergent genes further showed that they are enriched with transcription factor binding sites known to be linked to hematopoiesis, tumorigenesis, cell cycle, and development, suggesting the utility of systems level analysis for deriving valuable molecular level insights.

It is worth noting that our study suffers from a number of inherent limitations. With our attractor-based experimental setup, we could not detect early onset genes that lead to the "precommitment" state. Since gene expression profiles are only recorded after the two populations of the cells have achieved similar percentages of CD11b expression, prior cellular events of interest that culminate in their perspective promyelocyte and neutrophil attractors would be missed. In addition, since our study is based on a population of cells, inherent to all microarrays studies, only measurements of the average cellular behavior are possible. Indeed, it is known that the expression kinetics on a single cell level can exhibit an all-or-none switch like behavior, unlike the seemingly gradual change of expression when measured as a population average [39]. Further, recent evidence now suggests that transcriptional noise inherent to individual cells underlies clonal heterogeneity [83]. In light of this, an analysis on the gene expression changes of individual cells flowing toward the promyelocyte and neutrophil attractors would provide valuable insights.

Our study also suggests a number of natural extensions. For example, since transcription factor binding sites frequently occur in clusters and exert their effects simultaneously, instead of looking for enrichment of individual transcription factors, one can investigate enrichment of multiple transcription factors. Another possible extension is the incorporation of protein-protein interaction networks in order to identify potential co-activators of the transcriptional complexes governing HL60 differentiation. Further, one can search for common interaction partners to multiple enriched transcription factors. Additionally, in our characterization of divergent genes, comparisons of gene expression were made on a daily basis. To mitigate the effects of measurement noise and daily fluctuations, it is possible to model the entire time course gene expression profile for each gene by fitting a regression curve, promoting a possibly more robust identification of divergent genes.

Our study also raises an important question – can the concept of cell fate be sufficiently described by the use of one or few markers? Traditionally, cell fate has been intimately tied to the expression of cell surface receptors. However, in our study, two populations of ATRA-stimulated HL60 cells can both exhibit characteristic cell fate markers, yet be destined to have distinct cell fates, namely a promyelocyte or a neutrophil. This suggests two populations of cells may have the same "apparent" state as measured by cell surface markers while differing in other state space dimensions, ultimately leading them to disparate cell fates. Likewise, Chang *et al*. [39] also came to similar conclusions in their observation that low-dose DMSO-treated CD11b- cells are in a "primed" differentiated state as compared to DMSO-untreated CD11b- cells.

This study suggests that systems-level dynamics, such as the partitioning of the state space into distinct basins of attraction, have the potential to convey information about the molecular-level control of biological processes.

## Methods

### Cells and chemicals

Early passaged HL60 cells were generously provided by Dr. Steven Collins (Fred Hutchinson Cancer Research Center). The cells were cultured in T/25 flasks with RPMI-1640 media (Gibco) supplemented with 10% heat-inactivated fetal calf serum (Sigma). Cells were grown in media containing All-Trans Retinoic Acid (Sigma) to induce neutrophil differentiation [33]. ATRA was stored in -30°C and dissolved in 95% ethanol to make stock solution.

### Dosage and duration of ATRA treatments

A two-factor dosage and duration experiment was set up with eight and ten levels, respectively. HL60 cells were subjected to the following dosages (*μ*M): 1, 0.5, 0.1, 0.05, 0.01, 0.005, 0.001, and 0.0005 in conjunction with the following durations (Days of Treatment): 4, 5, 6, 7, 8, 9, 10, 11, 12, and 13. For example, one of the eighty treatment combinations is 1 *μ*M/4 Days, in which, we treated the HL60 cells with 1 *μ*M of ATRA for 4 Days and measured the CD11b expression of these treated cells by flow cytometry. Each of the 80 dosage/duration combinations and CD11b measurements were performed in triplicate.

### Flow cytometry to detect surface CD11b expression

Surface CD11b is an early marker of neutrophil differentiation in HL60. Cells (10^6^) were harvested, washed twice with PBS buffer, and incubated on ice for an hour with PE-conjugated CD11b antibody or its isotype control (Mouse IgG1*κ*) from BD Pharmingen. Cells were then washed two more times with PBS buffer and fluorescence was measured by FACSCalibur using CellQuest software (BD Biosciences). The CD11b expression levels were compared to the isotype control to correct for any non-specific binding. Untreated HL60 cells stained with the isotype control were used as background for undifferentiated cells.

### Dosage and duration contour plot construction

One million cells were collected for each experiment. Percentages of CD11b+ expression were calculated by comparing ATRA treated HL60 samples to untreated samples. Triplicate values of the percentage of differentiation were averaged and the result was displayed as a contour plot (Figure 1). In constructing the dosage/duration contour plot, one round of linear interpolation was performed to obtain a finer sampling of the dosage/duration grid.

### Wright-Giemsa stain to observe nuclear morphology

Treated and untreated cells (10^5^) were harvested, washed once with PBS buffer, spun down on microscope slides using Cytospin 2 (Shandon Inc.) and air-dried. Slides were then flooded with 2 mL Modified Wright-Giemsa stain (Sigma-Aldridge) and soaked for 1 minute. 2 mL deionized water was added, the slides were soaked for 3 minutes, rinsed with deionized water, and air dried.

### Microarray experiment and analysis to measure changes in mRNA levels

Two treatment combinations that yielded similar levels of CD11b expression were identified from the dosage and duration contour plot: 1) high dosage/short duration (1 *μ*M ATRA/5 Days) and 2) low dosage/long duration (0.05 *μ*M ATRA/7 Days). HL60 cells were cultured under these two conditions and fifteen million CD11b positive cells were collected from each condition using the Cytopeia inFlux V-Gs high-speed sorter. Sorted CD11b positive cells were then re-cultured in fresh ATRA-free RPMI media. After six hours of allowing these cells to recover from the sorting process, one million cells were collected. For the next five days, one million cells were collected every twenty-four hours, culminating in a total of 5 time points – Day 0 (6 hours post-sorting) to Day 4 (102 hours post sorting). For each time point, total RNA from cell samples were isolated with Trizol and quantified using Thermo Scientific NanoDrop 1000. RNA quantity for three technical replicates was collected for each time point, except day0, day1, and day4 of the 1 *μ*M treatment, where unfortunately the RNA quantity was only sufficient for two replicates, resulting in a total of 27 microarray experiments on Agilent human whole genome oligo arrays with 44 k 60-mer probes. ATRA-treated samples from each time point were labeled with the Cy5 dye, while untreated HL60 cells were labeled with the Cy3 dye for comparison. Each hybridized array was scanned with the Agilent dual laser-based scanner. Feature Extraction software version 8.0 (Agilent Technologies) was used to output the relative fluorescence intensity between the treated and untreated samples.

After the microarray experiments, the slides were scanned and the raw spot intensity (gProcessedSignal and rProcessedSignal from the feature extraction software) were used for subsequent data analysis in Matlab (The Mathworks, Inc). Of the 43931 spots on the Agilent array, spots that were designated for quality control, spots that were saturated, and spots that had signals too low to be detected were filtered out, resulting in 41509 spots remaining. The log intensity values were normalized using quantile normalization [84]. Replicate array intensity values were averaged to obtain the mean fluorescence intensity. Differentially expressed genes were picked using SAM [85] (Two class Paired). After this filtering step, 14949 differentially expressed genes remained. We compared the expression of 1 *μ*M ATRA/5 Days treatment with 0.05 *μ*M ATRA/7 Days treatment to identify genes that showed a steady daily increase of divergence in expression of 5% or larger, and designated these as divergent genes. Of these genes, those with elevated (respectively, suppressed) expression under both 1 *μ*M ATRA/5 Days treatment and 0.05 *μ*M ATRA/7 Days treatment relative to the untreated condition during the first three time points were deemed to be up-regulated (respectively, down-regulated). Comparisons of expressions were done on a daily basis, yielding a total of 176 divergent probes, 48 up-regulated and 128 down-regulated. A complete list of divergent gene probes is available in the supplementary section. Gene probes are sorted based on log_2 _fold divergence of Day 4 expression between 1 *μ*M ATRA/5 Days treatment and 0.05 *μ*M ATRA/7 Days treatment. The 176 divergent spots identified corresponded to 154 unique genes, 48 up-regulated genes and 106 down-regulated genes (some genes have multiple probes on the array).

### Functional enrichment of divergent genes by Gene Ontology (GO)

Up-regulated divergent genes and down-regulated divergent genes were submitted separately to GoMiner [62] for an analysis of enriched biological processes at the *p*-value level of 0.05 and FDR of 0.05.

### Searching for enriched transcription factor binding sites

Upstream 2 kb promoter regions of the divergent genes were downloaded from EMBL (NCBI36) using the BioMart interface [86]. Prepackaged upstream 2 kb regions of all RefSeq genes were also downloaded using the UCSC genome browser (hg18) [87]. After filtering out duplicated sequences and sequences containing ambiguous nucleotide bases, 18827 sequences remained. 429 human TRANSFAC (Professional 9.4) matrices [88] were used to calculate the log likelihood ratio scores of transcription factor binding for the divergent genes as well as the RefSeq genes, as

(1) $$L=\sum_{i=1}^{n}\log(P_{i}(M_{s}|s_{i}))-\sum_{i=1}^{n}\log(P_{i}(M_{b}|s_{i}))$$

where *L *is the log likelihood ratio score, *M*_*s *_is the TRANSFAC matrix model, *M*_*b *_is the zeroth order Markov background model with frequencies of A:0.2583 C:0.2457 G:0.2425 T:0.2535, which were calculated by counting the occurrences of the nucleotides in all promoter sequences. *s*_*i *_is the *i*th nucleotide of the motif site under consideration, and *n *is the length of the motif site.

Since binding sites tend to occur in clusters in higher eukaryotes [89], attention was paid to find stretches of the DNA sequences (100 bp) with large numbers of putative binding sites. Hence, log likelihood ratio scores from all 429 TRANSFAC matrices were summed at each nucleotide position for all RefSeq promoter sequences. The top 1% of these 100 bp highest scoring regions were picked as cut-off values to represent regions with clusters of putative binding sites. This cut-off value was then used to search for clusters of putative binding sites in the divergent genes, resulting in 262 of these clusters of binding sites being identified for the divergent genes. Expected numbers of binding sites were calculated by counting the total number of binding sites within the high-scoring regions divided by the total number of high-scoring regions for each transcription factor. This calculation was repeated for each TRANSFAC matrix. Enriched transcription factor binding sites were then ranked by the differences between the expected values for the divergent gene set and the set of all RefSeq promoters (Table 2).

## Authors' contributions

Conceived and designed the experiments: AH, SK, WZ, IS. Performed the experiments: AH, LH. Analyzed the data: AH. Contributed reagents/materials/analysis tools: WZ, IS. Wrote the paper: AH, IS.

## Supplementary Material

Additional file 1 **Numerical CD11b expression of ATRA-treated HL60 cells.** This file contains the underlying numerical values of the CD11b expression presented in the contour plot figure. Click here for file

Additional file 2 **List of divergent genes along with magnitude of divergence.** This file contains the complete list of identified divergent genes along with magnitude of log2 fold divergence. Click here for file
